# Great Achievements under Suffering

**Published:** 1893-09-30

**Authors:** 


### GREAT ACHIEVEMENTS UNDER SUFFERING.

### IV.—THE BLIND OBSERVER.

There are few lives more touching in completeness and content than that of Francis Huber. The serenity which marks its course is perhaps more apparent from the scantiness of any record of his personal experience, and yet every word in the beautiful little memoir of him, written by his friend, De Candolles, shows him to have been one of those natures born with the secret of living—the art of so adapting his will to his powers that every accident of life should work for good.

Born at Geneva, in 1750, he inherited from his father scientific perceptions which early urged him to incessant activity of mind. One of his uncles had ruined himself in the search for the philosopher's stone, and the young Huber spent long hours in his laboratory, drinking in with much curious and useless lore that love for science which proved of magic power through the long years of his deprivation to raise him above regret and vexation of spirit, and turn all his life to gold. His keenness of observation and delight in nature were early remarkable, and his restless eagerness in acquiring knowledge seemed a kind of instinct, leading him to store up recollections and feelings as a boy to last him all his life. Studying hard all day, and reading romances late into the night, often by moonlight, his eyesight together with his general health began by the age of fifteen to be impaired. He was taken to Paris to consult the oculist Venzel, who gave him from the first no hope of cure. Cataract was spreading over one eye, and the operation of couching being at that time extremely hazardous, Venzel could predict only total blindness. The other eye was affected then or later with "gutta serena"; Milton's "so thick a drop serene." Even many years after, when it seemed probable that an operation would restore him to partial sight, Huber would never entertain the idea.

There is no record as to the effect on his mind of the knowledge of his fate. To restore his general health, much enfeebled by study, he was sent for a few months to live the life of a peasant in a village near Paris, and here, no doubt, while following the plough and learning to love the country, he looked the future in the face. He had always a tender recollection of this pause in his life. He left it to enter upon a period of acute anxiety and pain. In the quaint words of his biographer, "His eyes, notwithstanding their weakness, had before his departure, and after his return, met those of Maria Aimée Sullin, a daughter of one of the syndics of the Swiss Republic." They had been attached to each other from childhood, and "neither of them thought it possible that anything could separate them." The attempt was made, however, by M. Sullin, who refused ever to consent to his daughter's marriage with a blind man. He succeeded in delaying their union for seven years, until which time, when Maria was twenty-five, she could not be lawfully married against his will. During all this time the cataract was slowly extending until every gleam of light was shut out. In the dread of losing his fiancée, Huber learnt to hide his increasing blindness long after all clear vision had abandoned him, and the habit clung to him all his life of commenting on the beauty of a landscape he knew by recollection, or "the fair complexion of a female whose voice pleased him." The wedding took place at last on the bride attaining her majority, and from that time till her death, forty years later, her devotion more than atoned to her husband for his missing sense. "As long as she lived," he could write in his old age, "I was not sensible of being blind."

Mme. de Stael has left in Delpine (Part III., Letter 19) a charming picture of the Huber family under the name of the Belmonts. "M. de Belmont interested us every moment, more by his wit and sterling sense. We induced him to speak of his occupations and interests. He replied always with pleasure, appearing to forget completely that he was blind and ruined, and giving the idea of a happy and peaceful being who has not the slightest occasion in life to exert courage or even resignation. Only in pronouncing the name of his wife, in calling her 'my dear friend,' there was a tone which I cannot define, but which echoed to all the memories of his life, and made them known to us without need of further words."

Music was always one of his principal recreations; and so active a mind could never have lacked interests even in poverty and isolation. But chance at length brought him under the influence of that curious power of attraction which bees possess for their friends and return in kind. After reading all that was then known on the subject, he became sceptical about some of the facts of their history, and intensely curious to account for others. He began to train an assistant, in the person of his young servant Francis Burnens, and by patient teaching and a special skill in questioning, which remained a noteworthy trait in his conversation, he so sharpened his powers of observation, that he could draw from him the smallest points relating to the structure and habits of the insects. He verified these observations by comparing them with those of his wife and friends, and thus formed in his own mind a perfect image of the minutest facts. "I am much more certain," he told his friend De Candolles, one day with a smile, "of what I perceive than you are, for you publish what your own eyes only have seen, while I take the mean among many witnesses." He was led by the difficulty of penetrating the secrets of the ordinary hives to invent a variety of new kinds in glass, which enabled him to watch every detail of the economy of the bees' kingdom. He published the result of his researches in 1792 in a volume called *New Observations on Bees*, which made a great stir in the scientific world, especially when the author's deprivation of sight became known. He was admitted into most of the learned academies of Europe, and though made at a time when scientific methods were in their infancy, no error has ever been detected in the delicate observations which he records.

Through all the stormy times of the Revolution, when his affairs became seriously embarrassed and his faithful assistant, Burnens, was removed from him, he continued his labour of love, aided now by his son. He turned his investigations next on the origin of the wax, a point which had hitherto been much disputed, and made elaborate researches into the construction of the cells, and the part taken by each class of bee in forming the hive. He was much puzzled by the problem of the respiration of bees, and the apparent impossibility of the oxygen being renewed in the interior of the hive. It had been too easily assumed by naturalists that bees could live without oxygen, but after many experiments Huber satisfied himself that the bees renewed the air by shaking their wings in a particular manner, somewhat after the fashion of an Indian punkah.

His literary style is remarkably picturesque and vivid, and gives the impression that the facts recorded are actually present to the eye. The labour and method, in fact, which enabled him to form a clear conception in his own mind, sufficed to extend it to his readers.

In his domestic life he was singularly happy. "One thing I have never been able to learn," he said in extreme old age, "that is, to forget how to love." He was surrounded by friends, whose attachment strengthened with years, and his kindliness, humour, and keen perceptions made him a charming companion to young and old. The animation of his face, and in particular of his eyes, when any subject specially interested him caused the remark in a celebrated savant, who saw him for the first time, that he could now understand why the blind were thought to be supernaturally inspired.

He outlived his wife for some few years, which were passed under the care of his daughter at Lausanne, and died in 1831 at the age of eighty-one, having retained all his faculties to the last. "He was loving and beloved to the end of his days." What higher blessing could sight have lent to his life?

